# Common Patterns and Unique Threats in Antimicrobial Resistance as Demonstrated by Global Gonococcal Surveillance 

**DOI:** 10.3201/eid3014.240296

**Published:** 2024-11

**Authors:** Adriana Le Van, Nazia Rahman, Reuel Sandy, Nelson Dozier, Hunter J. Smith, Melissa J. Martin, Katelyn V. Bartlett, Krit Harncharoenkul, Anna Nanava, Tamar Akhvlediani, Paul Rios, Supriya D. Mehta, Walter Agingu, Denis K. Byarugaba, Fred Wabwire-Mangen, Hannah Kibuuka, Bernard Erima, Hope O. Kabatasi, Naiki Attram, Dutsadee Peerapongpaisarn, Wilawan Oransathit, Wirote Oransathit, Umaporn Suksawad, Woradee Lurchachaiwong, Somchai Sriplienchan, Nonlawat Boonyalai, Maneerat Somsri, Nithinart Chaitaveep, Ann Jerse, Eric Garges

**Affiliations:** Henry M. Jackson Foundation for the Advancement of Military Medicine, Bethesda, Maryland, USA (A. Le Van, R. Sandy); Uniformed Services University, Bethesda (A. Le Van, R. Sandy, N. Dozier, A. Jerse, E. Garges); Cherokee Nation Strategic Programs, Tulsa, Oklahoma, USA (N. Rahman); Global Emerging Infections Surveillance Branch, Armed Forces Health Surveillance Division, Silver Spring, Maryland, USA (N. Rahman, H.J. Smith); Multidrug Resistant Organism Repository and Surveillance Network, Walter Reed Army Institute of Research, Silver Spring (M.J. Martin, K.V. Bartlett); Walter Reed Army Institute of Research, Armed Forces Research Institute of Medical Sciences, Bangkok, Thailand (K. Harncharoenkul, D. Peerapongpaisarn, Wilawan Oransathit, Wirote Oransathit, U. Suksawad, W. Lurchachaiwong, S. Sriplienchan, N. Boonyalai, M. Somsri, N. Chaitaveep); Walter Reed Army Institute of Research Europe-Middle East, Tbilisi, Georgia (A. Nanava, T. Akhvlediani); US Naval Medical Research Unit SOUTH, Lima, Peru (P. Rios); Rush University College of Medicine, Chicago, Illinois, USA (S.D. Mehta); Walter Reed Army Institute of Research-Africa, Kisumu, Kenya (W. Agingu); Makerere University Walter Reed Project, Kampala, Uganda (D.K. Byarugaba); Makerere University College of Veterinary Medicine, Kampala (D.K. Byarugaba, F. Wabwire-Mangen, H. Kibuuka, B. Erima, H.O. Kabatasi); US Naval Medical Research Unit EURAFCENT, Accra, Ghana (N. Attram); Walter Reed Army Institute of Research-Africa, Nairobi, Kenya (E. Garges)

**Keywords:** bacteria, sexually transmitted infections, *Neisseria gonorrhoeae*, gonorrhea, surveillance, antibiotic resistance, gonococci, antimicrobial resistance, Global Emerging Infections Surveillance, GEIS, global, United States

## Abstract

The rapid emergence of antimicrobial-resistant strains of *Neisseria gonorrhoeae* threatens treatment options and control efforts. The Uniformed Services University Gonococcal Reference Laboratory and Repository of the Global Emerging Infections Surveillance Program receives isolates from several geographically distinct regions worldwide. We analyzed 962 isolates collected during 2014–2022 for genomic and phenotypic antimicrobial resistance. Resistance to antimicrobial drugs previously used for gonococcal infections was high, but of most concern were increases of resistance to currently used antibiotic drugs, such as extended-spectrum cephalosporins and the alternative antibiotic treatment gentamicin. The percentage of isolates with reduced susceptibility to ceftriaxone was 3.6%, to cefixime was 2.5%, and to gentamicin was 15.0%. Although isolates were collected from populations of limited diversity, 706 (73.4%) of isolates demonstrated novel multiantigen sequence types, and 225 (23.4%) had novel multilocus sequence types. Continued surveillance of *N*. *gonorrhoeae* is essential to monitoring the prevalence and spread of resistant organisms worldwide.

*Neisseria gonorrhoeae* infections cause substantial illness globally, and control is challenged by increasing antimicrobial resistance. The World Health Organization (WHO) reported 82.4 million new *N*. *gonorrhoeae* infections worldwide among persons 15–49 years of age (*1*). In the United States, an estimated 1.5 million new cases of gonorrhea are reported each year (*2*).

Gonococcal urogenital tract infections can cause severe complications, especially in women, who are often asymptomatic and go undiagnosed. Untreated cervical infections can cause upper genital tract disease, such as pelvic inflammatory disease, chronic pelvic pain, and ectopic pregnancy, and also increases the risk for tubal infertility. Urethral infections in men can ascend to cause epididymitis or orchitis; however, unlike cervical infections, urethral infections are usually symptomatic. The resulting discharge and dysuria increase the likelihood that male patients will seek testing and treatment.

Effective infection control is challenged by underdiagnosis of asymptomatic infections, lack of point-of-care diagnostics, and increasing persistent antimicrobial resistance. *N*. *gonorrhoeae* has developed resistance to all antibiotic drugs that have been used for routine treatment because of its ability to readily acquire genes through horizontal gene transfer or spontaneous mutations. The prevalence of antimicrobial resistance (AMR) within *N*. *gonorrhoeae* strains has steadily increased across the antibiotic era, necessitating frequent changes in treatment recommendations. The initial emergence of high-level penicillin and tetracycline resistance was followed by the introduction of fluoroquinolones for gonorrhea treatment in the mid-1980s, which were subsequently removed from treatment in 2007 (*3*). Dual therapy using extended-spectrum cephalosporins (ESCs) and azithromycin then became the primary recommended therapy for a decade. Azithromycin was removed in 2021 because of increasing resistance, leaving only ESCs for first-line treatment of gonorrhea. Globally, ceftriaxone is the sole remaining primary therapy for first-line treatment of gonorrhea in most guidelines (*4*–*6*). However, isolates with reduced susceptibility to ceftriaxone have proliferated worldwide, and multidrug-resistant, ceftriaxone-resistant strains have been reported in several countries (*7*–*10*), threatening simple outpatient therapy.

Because of the threat of untreatable gonorrhea, *N*. *gonorrhoeae* is classified by the Centers for Disease Control and Prevention as an urgent threat (*11*) and by WHO as a high-priority pathogen (*12*) for which new treatments are critically needed. Global rates of *N*. *gonorrhoeae* infections have been reported since 1992 through the WHO Enhanced Gonococcal Antimicrobial Surveillance Programme (EGASP). Data for 2017–2018 from 73 countries demonstrated resistance to ESCs of 0%–22%, azithromycin resistance of 0%–60%, and ciprofloxacin resistance of 0%–100% (*13*). Although several countries report AMR data to the EGASP, *N*. *gonorrhoeae* surveillance data from many global regions, such as Central America, Eastern Europe, Southeast Asia, sub-Saharan Africa, and the Eastern Mediterranean, remain scarce.

The Sexually Transmitted Infection (STI) National Strategic Plan for the United States (2021–2025) recognizes the need to improve STI prevention at the local, state, and federal levels. The plan also recommends that specific groups, such as the military and fraternal organizations, include services that address men’s sexual health and their role in transmitting STIs (*14*). Military service members are at high risk for STI because of social demographics including age; however, factors such as increased alcohol consumption, diversification of sexual networks, and infrequent condom use also exacerbate risk in military populations (*15*). In addition, sexual assault, which carries an inherent risk for STI, has been reported in 1.0% of men and 4.9% of women in military service (*16*).

In alignment with the National Action Plan for Combating Antibiotic-Resistant Bacteria (*14*), and to monitor this urgent, ever-changing AMR threat, the Uniformed Services University (USU), in collaboration with the Armed Forces Health Surveillance Division’s Global Emerging Infections Surveillance (GEIS) Branch, established the USU Gonococcal Reference Laboratory and Repository (GC Repository) within the USU Department of Microbiology and Immunology (Bethesda, Maryland, USA). This report analyzes trends in the susceptibility of *N*. *gonorrhoeae* isolates from different geographic regions to 8 different antibiotic drugs during 2014–2022 as part of the GEIS STI surveillance program. We also report the distribution of key alleles on the basis of genomic analysis to help define the prevalence of specific AMR determinants in different geographic regions. The investigators have adhered to the policies for protection of human subjects as prescribed in AR 70-25.

## Methods

The GEIS STI initiative was established in 2010 to improve the health of the US armed forces and support force health protection decision-making. The GC Repository was established in 2014 to serve as a central entity for confirmatory testing and both phenotypic and genotypic characterization (*17*–*19*). As part of the surveillance program, a proficiency testing program was also established for quality assurance of partner laboratory methods for *N*. *gonorrhoeae* AMR testing.

### Sampling Methods

We collected samples from persons enrolled in clinical care or public health surveillance activities during 2014–2022, which included military populations, civilians, and high-risk populations from 5 geographic regions. We gram-stained from urethral, vaginal, cervical, pharyngeal, or rectal swab samples, plated them on selective media such as modified Thayer-Martin agar, and incubated for 24 hours at 37°C in 5% CO_2_ or in a candle jar. We froze isolates of presumptive *N*. *gonorrhoeae* in 25% glycerol and tryptic soy broth and shipped to the GC Repository. We assessed AMR using Etest (bioMérieux, https://www.biomerieux.com) (Appendix) and performed agar dilution to confirm MICs for isolates with reduced susceptibility to azithromycin, ceftriaxone, cefixime, and gentamicin.

### Reference Laboratory Testing

As of December 2023, the GC Repository received a total of 1,244 presumptive isolates from 6 countries: Thailand (n = 557), the Philippines (n = 35), Ghana (n = 73), Peru (n = 237), Kenya (n = 211), and Georgia (n = 95). We confirmed isolates by culture on modified Thayer-Martin agar, Gram staining, oxidase test positivity, superoxol test positivity, and API NH biochemical test (bioMérieux). We used detection of the *porA* pseudogene to resolve inconclusive API NH test results (Appendix). We determined MICs for all 962 isolates (Appendix).

### Whole-Genome Sequencing and Bioinformatic Analysis

We sent *N*. *gonorrhoeae* isolates to the Walter Reed Army Institute of Research’s Multidrug-Resistant Organism Repository and Surveillance Network (Silver Spring, Maryland, USA) for whole-genome sequencing (Appendix) and genotypic characterization. Multilocus sequence typing (MLST) was performed in silico using the *N. gonorrhoeae* scheme curated by Maiden (*20*). We performed additional in silico molecular typing using *N. gonorrhoeae* multiantigen sequence typing (NG-MAST) and *N. gonorrhoeae* sequence typing for antimicrobial resistance (NG-STAR) with ngmaster version 1.0.0 (*21*) (Appendix).

## Results

Of the 1,244 frozen suspensions of presumptive *N*. *gonorrhoeae* from 5 geographic regions received by the GC Repository, 962 (77.3%) were confirmed as *N*. *gonorrhoeae* isolates. Among isolates for which the type of sample was recorded, most came from urethral swab samples taken from men. Limited, inconsistent demographic data were available to the partner laboratories involved in public health surveillance.

### Antimicrobial Susceptibility Testing

We compiled phenotypic AMR data for all 962 isolates (Table 1). Benzylpenicillin resistance was most commonly observed (917/962 [95.3%]), followed by tetracycline (902/962 [93.7%]) and ciprofloxacin (882/962 [91.7%]). Resistance to those antibiotic drugs varied among sites; ≈50% resistance to each of those antibiotic drugs was observed in Georgia, whereas other sites exhibited up to 90% resistance. Elevated MICs (IR>1; R>2) to azithromycin was found in 10 isolates, 8 of which had azithromycin MICs of 1 and 1.5 μg/mL (2 from Georgia, 2 from Peru, and 4 from Thailand); 2 isolates had MICs >256 ug/mL (Kenya). Among isolates from Kenya, 5 exhibited reduced susceptibility to the ESCs: 1 for cefixime, 2 for ceftriaxone, and 2 for both cefixime and ceftriaxone. Similarly, 11 Georgia isolates exhibited reduced susceptibility to ESCs. We observed that 84% (809/962) of the isolates were susceptible to gentamicin (S<4; IR = 8–16; R>32). Of the remaining isolates, 7 (0.7%) had MICs of 16 μg/mL (4 from Peru, 2 from Georgia, and 1 from Ghana). All 962 isolates were susceptible to spectinomycin. Multidrug resistance was common among all international collection sites. The frequency of resistance to any 3 antibiotic drugs ranged from 11% (Ghana) to 92% (Peru).

**Table 1:** Summary of phenotypic antimicrobial resistance in study of common patterns and unique threats in antimicrobial resistance as demonstrated by global gonococcal surveillance*

Region	Isolates with reduced susceptibility or resistance, no. (%)						
	Tetracycline	Benzylpenicillin	Ciprofloxacin	Azithromycin	Cefixime	Ceftriaxone	Gentamicin
Thailand, n = 516	500 (96.9)	502 (97.3)	500 (97)	4 (0.77)	2 (0.4)	16 (3.1)	31 (6.0)
Ghana, n = 19	19 (100)	19 (100)	17 (89.5)	0	1 (5.3)	0	7 (36.8)
Peru, n = 208	195 (93.7)	205 (98.5)	186 (89.4)	2 (0.96)	7 (3.4)	3 (1.4)	63 (30.3)
Nairobi, Kenya, n = 27	27 (100)	26 (96.3)	23 (85.1)	0	0	0	1 (3.7)
Kisumu, Kenya, n = 110	108 (98.2)	105 (95.5)	106 (96.4)	2 (1.8)	3 (2.72)	4 (3.63)	26 (23.6)
Uganda, n = 10	9 (90)	10 (100)	9 (90)	0	0	0	2 (20)
Georgia, n = 72	44 (61.1)	50 (69.4)	41 (56.9)	2 (2.8)	11 (15.2)	11 (15.2)	16 (22.2)
Total, N = 962	902 (93.7)	917 (95.3)	882 (91.7)	10 (1.02)	24 (2.5)	34 (3.6)	146 (15.2)

*MICs interpreted according to Clinical and Laboratory Standards Institute criteria when available (*22*). CLSI resistance breakpoints used for penicillin (I>0.06; R>2.0 μg/mL), tetracycline (I>0.25; R>2.0 μg/mL), and ciprofloxacin (I>0.06; R>1.0 μg/mL) (*22*). Gonococcal Isolate Surveillance Project breakpoints used for azithromycin (I>1; R>2.0 μg/mL), cefixime (I>0.06; R>0.25 μg/mL), and ceftriaxone (I>0.06; R>0.125 μg/mL) (*23**,**24*), because CLSI has not established criteria for resistance to those antimicrobial drugs. Gentamicin breakpoints (I≥8–16 μg/mL; R I>32.0 μg/mL) were determined according to research published by the Centers for Disease Control and Prevention (*25*).

### Molecular Determinants of AMR and Genomic Characterization

All 676 isolates with high-level tetracycline resistance (Tet^R^) (MIC >8 μg/mL) (676/962 [97.7%]) isolates) harbored the *tetM* gene and the *rpsJ* V57M mutation, whereas isolates with MICs of 0.5–3 μg/mL did not carry the *tetM* gene but had the *rpsJ* V57M mutation (Table 2). Among the 917 benzylpenicillin-resistant isolates carrying β-lactamase-producing plasmids, 4 different β-lactamase resistance genes were detected; *bla*_TEM-1_ was detected in 57.1% of isolates and *bla*_TEM-135_ was detected in 10.3% of isolates. One isolate from Peru harbored the *bla*_TEM-22_ plasmid. The *bla*_TEM-239_ plasmid was present in 6 isolates from East Africa (1 from Uganda and 5 from Kenya).

**Table 2:** Presence of antimicrobial-resistant genetic determinants in study of common patterns and unique threats in antimicrobial resistance as demonstrated by global gonococcal surveillance*

Region	No. (%) isolates												
	Tetracycline resistance			Benzylpenicillin resistance			Ciprofloxacin resistance			Azithromycin resistance		Cefixime and ceftriaxone resistance	
	V57	*tetM*		β-lactams	*ponA_L421P_*		*gyrA*	*parC*		*mtrD*		*Mtr*	*penA*
Thailand, n = 516	506 (98)	449 (87)		*bla*_TEM-1_, 327 (63.4); *bla*_TEM-135_, 77 (15)	80 (15.5)		S91, D95, 318 (61.6)	D86, 217 (42); S87, 218 (42.2); E91, 21 (4); S88, 8 (1.5)		*MtrD* S821A K823E, 7 (1.35)		Internal stop codon, 27 (5.3)	I312M V316T G545S, 2 (0.38)
Ghana, n = 19	19 (100)	15 (79)		*bla*_TEM-1_, 13 (68.4)	16 (84.2)		S91, D95, 17 (89.5)	D86, 3 (17.6); S87, 12 (70.6); E91, 1 (5.9)		0		G45D, 7 (36.8)	I312M V316T G545S, 1 (5.26)
Peru, n = 208	208 (100)	87 (41.8)		*bla*_TEM-1_, 111 (53.3); *bla*_TEM-135_, 13 (6.25); *bla*_TEM-22_, 1 (0.48)	120 (57.6)		S91; D95, 188 (90.3)	D86, 52 (28); S87, 46 (24.7)		*MtrD* mosaic 2, *MtrR* mosaic 2, 2 (0.96)		G45D, 21 (10)	I312M V316T G545S, 17 (8.17)
Nairobi, Kenya, n = 27	27 (100)	23 (85.2)		*bla*_TEM-1_, 22, 81.5)	12 (44.4)		S91, D95, 21 (77.7)	D86, 1 (4.76)		0		0	0
Kisumu, Kenya, n = 110	110 (100)	99 (90)		*bla*_TEM-1_, 32 (29); *bla*_TEM-135_, 3 (2.72); *bla*_TEM-239_, 5 (4.54)	66 (60)		S91, D95, 106 (96.3)	D86, 10 (9); S87, 9 (8.2); E91, 65 (59)		23s rDNA A2045G, 2 (1.8)		A39, 84 (76.5); G45, 2 (1.8); D79, 11 (10); M197, 1 (0.9)	A501, F504, A516 N512, 4 (3.6)
Uganda, n = 10	10 (100)	10 (100)		*bla*_TEM-1_, 8 (80); *bla*_TEM-135_, 1 (10); *bl*a_TEM-239_, 1 (10)	5 (55)		S91, D95, 10 (100)	D86, 4 (40); S87, 2 (20); E91, 4 (40)		0		A39, 8 (80); D79, 2 (20)	F504, 10 (100); A516, 10 (100)
Georgia, n = 72	47 (65.3)	14 (19.4)		*bla*_TEM-1_, 11 (15.3)	40 (55.5)		S91, D95, 41 (57)	D86, 12 (19.5); S87, 31 (75.6); E91, 12 (29.3)		*MtrD* mosaic 2, *MtrR* mosaic 2, 2 (2.7); *MtrD* S821A K823E, 5 (6.9)		A39, 28 (38.9); G45, 6 (8.3); D79, 14 (19.4); M197, 2 (2.7)	I312M V316T G545S, 9 (12.5%)
Total, N = 962	928 (96.4)	697 (72.5)		*bla*_TEM-1_, 524 (54.5); *bla*_TEM-135_, 94 (9.77); *bla*_TEM-239_, 6 (0.62); *bla*_TEM-22_, 1 (0.10)	339 (35.2)		S91, D95, 701 (72.9)	D86, 299 (31); S87, 318 (33); E91, 103 (10.7)				A39, 641 (66.6); G45D, 36 (3.74)	

*Percentages calculated on total number of *Neisseria gonorrhoeae* isolates confirmed for the country.

In contrast, the number of isolates harboring chromosomally mediated determinants of AMR varied widely. Mutations in the *mtrR* gene (G45D) were present in 3.7% of isolates, and mutations in the *mtr* promoter region (−35Adel) were present in 10% percent of isolates, whereas the A39THTH mutation was more prevalent (66.6% of isolates). Overall, we identified MtrR disruptions in 12% of isolates. The *ponA*L421P mutation was found in 35.2% of isolates, whereas *porB* mutations A121N, G120K, and A121D were less common and found in 2.2% of isolates (A121N), 8.6% of isolates (G120K), and 3.8% of isolates (A121D). All isolates with reduced susceptibility or resistance to ciprofloxacin (MICs 1 to >32 μg/mL) harbored S91F and D95G/A/N mutations in *gyrA*. Mutations in *parC* (D86, S87, or E91K) were found in 74.8% of isolates. We found the *fusA* A563V mutation, which confers reduced susceptibility to gentamicin, in 1 isolate from Peru (*26*,*27*).

Isolates with reduced susceptibility to azithromycin harbored myriad chromosomal resistance determinants. Mosaic *mtrD* and *mtrR* alleles were found in 4 isolates (2 from Georgia and 2 from Peru). One of those Georgia isolates also carried the *penA* mosaic allele XXXIV. One isolate from Peru carried the *mtrR* mosaic allele but lacked the *mtrD* mosaic allele. We found that 7 isolates from Thailand harbored *mtrD* S821A K823E mutations associated with azithromycin resistance (*28*), but only 4 of the 7 isolates had reduced susceptibility to azithromycin (MICs >1 μg/mL). The 23s rDNA A2045G mutation was present in 2 isolates from Kenya (MIC >256 μg/mL). Examination of ESC resistance determinants showed that 32 of the 962 isolates carried mosaic *penA* alleles. We detected 4 mosaic *penA* alleles: XXXIV (24 isolates [2.5%]), 166 (2 isolates [0.2%]), and 217 (5 isolates [0.5%]); 1 isolate (0.1%) had a novel allele. Of those 32 isolates, 29 had reduced susceptibility to cefixime and 11 had reduced susceptibility to both cefixime and ceftriaxone. We found that 5 isolates with reduced susceptibility to both ceftriaxone and cefixime did not carry a mosaic *penA* allele.

We monitored the type of *porB1* allele present, which encodes the major outer membrane porin (PorB). *N*. *gonorrhoeae* strains express 1 of 2 *porB1* alleles. The *porB1A* allele is associated with strains that cause disseminated infection, whereas strains with *porB1B* more frequently cause localized infections (*29*). Although *porB1B* strains are usually more common, the frequencies of *porB1A* and *porB1B* alleles were similar among the 962 isolates, except for Thailand, Georgia, and Peru isolates. Thailand isolates cultured before 2016 (n = 88) carried *porB1A* more frequently (81/88 [92.0%]) than *porB1B* (7/88 [8.0%]). After 2016, *porB1B* strains were isolated more often in Thailand. Among isolates from Georgia, only 5 (6.9%) isolates expressed *porB1A*, and in Peru, 62 (47.3%) of 131 *porB1A*-expressing isolates were collected during 2014–2017, compared with 14 isolates (18.2%) of 77 *porB1A* strains collected during 2018–2022.

**Figure 1:**
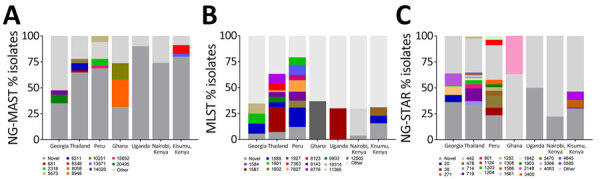
Distribution of most prevalent NG-MAST, MLST, and NG-STAR schemes in Global Emerging Infections Surveillance isolates of *Neisseria gonorrhoeae* received at Uniformed Services University, Bethesda, Maryland, USA, from sites outside the United States (n = 962) in study of common patterns and unique threats in antimicrobial resistance as demonstrated by global gonococcal surveillance. A) Percentage of isolates assigned to the most common NG-MAST types in each region. B) Percentage of isolates assigned to the most common MLST types in each region. C) Percentage of isolates assigned to the most common NG-STAR types in each region. MLST, multilocus sequence typing; NG-MAST, *N. gonorrhoeae* multiantigen sequence typing; NG-STAR, *N. gonorrhoeae* sequence typing for antimicrobial resistance.

**Figure 2:**
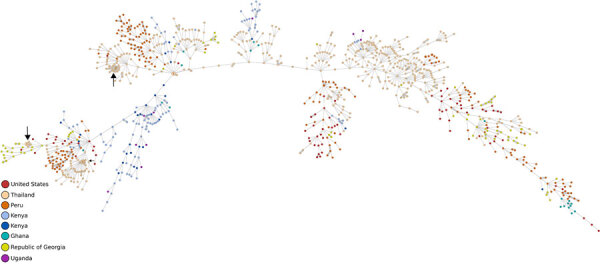
Minimum-spanning tree showing genome-based genetic relatedness of all *N. gonorrhoeae* isolates received at Uniformed Services University (n = 1,044), Bethesda, Maryland, USA, in study of common patterns and unique threats in antimicrobial resistance as demonstrated by global gonococcal surveillance. Tree was generated using core genome multilocus sequence typing. Each circle represents >1 isolates; isolates with 1–10 allelic differences are emphasized by gray shading around the lines and are considered highly genetically related with suspicion of nosocomial origin. Isolates are colored corresponding to their country of origin. Possible clonal isolates are shown with black arrows.

Molecular typing identified 98 NG-MAST, 198 MLST, and 199 NG-STAR sequence types (STs) among the 962 isolates (Figure 1). We found that 706 isolates belonged to a novel NG-MAST ST. The most common defined NG-MAST STs were ST6211 (n = 36), ST8058 (n = 21), ST2318 (n = 14), ST5573 (n = 12), and ST681 (n = 10). We identified novel MLST STs in 225 of 962 isolates. The most common MLST STs were ST1587 (n = 133), 1588 (n = 80), 7363 (n = 55), 8756 (n = 55), 8143 (n = 44), and 7827 (n = 40). Those isolates were all ciprofloxacin resistant. Using NG-STAR, we identified 173 novel types. The most common defined NG-STAR STs were ST719 (n = 69), ST271 (n = 24), ST801 (n = 23), and ST1203 (n = 22). The distribution of NG-MAST, MLST, and NG-STAR STs also revealed that certain STs are specific to various regions (Figure 1). We generated a minimum-spanning tree on the basis of core genome MLST of all isolates, categorized by geographic location, to examine genomic diversity and possible clonal spread (Figure 2). Isolates from Thailand clustered into 4 major groups, and 3 appear to be clonal isolates (black arrows). Georgia isolates also clustered, but some were closely related to isolates from Thailand (≈300 core genome allele differences). Isolates from Peru grouped into 5 clusters.

## Discussion

Increasingly resistant *N*. *gonorrhoeae* infections present a major public health burden for civilian communities, military force health protection, and US military readiness. Surveillance programs incorporating specimen culture are critical for linking genotypic and phenotypic AMR data to enable AMR prediction. The WHO EGASP program provides data from 68 countries in 6 regions as of 2018 (*30*). The GEIS network fills some key gaps in surveillance, including Eastern Europe and East Africa, where *N*. *gonorrhoeae* AMR data remain scarce.

This study reports phenotypic and genotypic analyses of geographically and temporally diverse NG isolates collected through the GEIS STI surveillance program. Isolates from international sites displayed high frequencies of resistance to benzylpenicillin, tetracycline, and ciprofloxacin, ranging from 50% to 100%. Those data are similar to data from previously published literature. Investigators from Peru identified ≈95% resistance to ciprofloxacin (*31*–*33*), whereas reduced susceptibility or resistance to penicillin was observed in 99.4% of isolates and to tetracycline in 94.5% of isolates (*31*). In Peru, 76% of isolates were reported to have reduced susceptibility to gentamicin (*31*). In comparison, however, our study identified reduced susceptibility to gentamicin in ≈15% of isolates. Regional differences in AMR patterns can be driven by community-based factors, including limited access to care and lack of available diagnostics, leading to empiric treatment. Similarly, lack of access to recommended antibiotic drugs and readily available access to other over-the-counter antibiotic drugs in the absence of valid healthcare encounters can also drive selection for *N*. *gonorrhoeae* AMR. For example, ciprofloxacin is still used empirically to treat STIs in Peru and other countries in Latin America. In Uganda, cefixime therapy is recommended but not easily available (*34*).

In Georgia, the recommended treatment for *N*. *gonorrhoeae* remains 1 g ceftriaxone plus 2 g azithromycin. Isolates from Georgia displayed lower frequencies of resistance to penicillin, tetracycline, and ciprofloxacin (≈50%) than did isolates from Africa and Asia (>90%). However, isolates from Georgia were more likely to exhibit reduced susceptibility to ESCs (≈15%) than were isolates from Asia and Africa (≈3.6%). *N*. *gonorrhoeae* can develop resistance to antibiotic drugs within a few decades of introduction (*35*). Earlier uptake of ESCs in Georgia might account for the decrease in susceptibility seen, compared with our isolates collected from the global south. Antimicrobial susceptibility among Georgia isolates might also be affected by population changes caused by neighboring political unrest. Several studies of STIs in migrants, refugees, and internally displaced persons observe that these populations might be at higher risk for sexual assault and STI (*36*). However, the potential association between migration and *N*. *gonorrhoeae* AMR requires further study (*37*).

Many multidrug-resistant *N*. *gonorrhoeae* isolates originate in Asia (*1*). However, isolates from Thailand tested at the GC Repository exhibited low overall frequencies of resistance to primary therapies such as cefixime (0.4%), ceftriaxone (3%), and azithromycin (0.77%). The findings are surprising given the regional history of resistant *N*. *gonorrhoeae*; however, other recent surveillance studies in Thailand have observed similar results (*38*). The GC Repository recently received 18 isolates collected from high-risk patients in Pattaya, Thailand, that exhibited higher frequencies of resistance to macrolides and ESCs.

Recently, the US Centers for Disease Control and Prevention published guidelines on preventive treatment for bacterial STIs using doxycycline postexposure prophylaxis (doxyPEP) (*39*). Multiple prospective studies observed a reduction in incident bacterial STIs among men who have sex with men who were taking doxyPEP (*40*–*42*). Those studies have largely focused on syphilis, but the effect on *N*. *gonorrhoeae* infection has been noted. For example, in South Africa, doxyPEP reduced *N*. *gonorrhoeae* infections in men by 50%, but no difference was observed in cisgender women in Kenya taking doxyPEP compared with women in the standard care group (*43*). Many isolates tested at the GC Repository had the *tetM* gene, which is harbored in the easily spread pCONJ plasmid and can be transferred with *pbla* (*44*,*45*), which might counter the potential effectiveness of doxyPEP for gonorrhea prevention. Although doxycycline therapy is not commonly used for contemporary treatment of *N*. *gonorrhoeae*, continued surveillance is essential to understand the potential effects of doxyPEP on transmission and AMR.

Limitations of this study include low sample size and a study population that might be neither population-representative nor representative of the United States or partner nation militaries. As previously mentioned, most isolates originated from urethral samples taken from men, largely because of both local clinical standards of care at collection sites and ease of sample collection and culture. Genital specimens from female patients, in contrast, are more difficult to culture, possibly because of the robust female urogenital microbiome. Extragenital isolates, which were infrequent in this study, are particularly relevant because of their proximity to commensal *Neisseria*, which may provide opportunities for horizontal gene transfer and acquisition of genetic determinants of AMR. In addition, the GC Repository has limited access to demographic and clinical data, such as sex or military status.

As of January 2024, two new antibiotic drugs for the treatment of gonorrhea infections, zoliflodacin and gepotidacin (*46*,*47*), have undergone Phase III clinical trials with promising results. Even with impending availability, however, the ease of AMR development in *N*. *gonorrhoeae* still portends a grim outlook for long-term treatment effectiveness. Without a vaccine, enhanced surveillance of *N*. *gonorrhoeae* AMR that combines culture, epidemiologic information, and molecular data must continue to identify genetic determinants of AMR and inform appropriate treatment recommendations.

Appendix Additional information about common patterns and unique threats in antimicrobial resistance as demonstrated by global gonococcal surveillance

## References

[1] World Health Organization. Multi-drug resistant gonorrhoea [cited 2024 Jul 4]. https://www.who.int/news-room/fact-sheets/detail/multi-drug-resistant-gonorrhoea

[2] Kreisel KM, Spicknall IH, Gargano JW, Lewis FMT, Lewis RM, Markowitz LE, et al. Sexually transmitted infections among US women and men: prevalence and incidence estimates, 2018. Sex Transm Dis. 2021;48:208–14. 10.1097/OLQ.0000000000001355 33492089 PMC10245608

[3] Centers for Disease Control and Prevention. CDC changes recommendations for gonorrhea treatment due to drug resistance [cited 2007 Apr 12]. https://archive.cdc.gov/www_cdc_gov/media/pressrel/2007/r070412a_1697124700.htm

[4] Kenyon C, Laumen J, Van Dijck C, De Baetselier I, Abdelatti S, Manoharan-Basil SS, et al. Gonorrhoea treatment combined with population-level general cephalosporin and quinolone consumption may select for Neisseria gonorrhoeae antimicrobial resistance at the levels of NG-MAST genogroup: An ecological study in Europe. J Glob Antimicrob Resist. 2020;23:377–84. 10.1016/j.jgar.2020.10.022 33207228

[5] Workowski KA, Bachmann LH, Chan PA, Johnston CM, Muzny CA, Park I, et al. Sexually transmitted infections treatment guidelines, 2021. MMWR Recomm Rep. 2021;70:1–187. 10.15585/mmwr.rr7004a1 34292926 PMC8344968

[6] World Health Organization. Guidelines for the management of symptomatic sexually transmitted infections. Geneva: The Organization; 2021. 34370424

[7] Unemo M, Golparian D, Nicholas R, Ohnishi M, Gallay A, Sednaoui P. High-level cefixime- and ceftriaxone-resistant *Neisseria gonorrhoeae* in France: novel *penA* mosaic allele in a successful international clone causes treatment failure. Antimicrob Agents Chemother. 2012;56:1273–80. 10.1128/AAC.05760-11 22155830 PMC3294892

[8] Day M, Pitt R, Mody N, Saunders J, Rai R, Nori A, et al. Detection of 10 cases of ceftriaxone-resistant *Neisseria gonorrhoeae* in the United Kingdom, December 2021 to June 2022. Euro Surveill. 2022;27:2200803. 10.2807/1560-7917.ES.2022.27.46.2200803 36398578 PMC9673238

[9] Kueakulpattana N, Wannigama DL, Luk-In S, Hongsing P, Hurst C, Badavath VN, et al. Multidrug-resistant *Neisseria gonorrhoeae* infection in heterosexual men with reduced susceptibility to ceftriaxone, first report in Thailand. Sci Rep. 2021;11:21659. 10.1038/s41598-021-00675-y 34737332 PMC8569152

[10] Centers for Disease Control and Prevention. AMR gonorrhea: two cases of concern identified [cited 2023 Jan 23]. https://archive.cdc.gov/#/details?url=https://www.cdc.gov/std/dstdp/dcl/2023-01-19-bachmann-amr-gonorrhea.htm

[11] Centers for Disease Control and Prevention. Antibiotic resistance threats in the United States, 2019 [cited 2019 Nov 13]. https://www.cdc.gov/antimicrobial-resistance/data-research/threats

[12] World Health Organization. Diagnostics for gonococcal antimicrobial resistance [cited 2023 Jan 23]. https://www.who.int/teams/global-hiv-hepatitis-and-stis-programmes/stis/testing-diagnostics/diagnostics-for-gonococcal-antimicrobial-resistance

[13] Unemo M, Lahra MM, Escher M, Eremin S, Cole MJ, Galarza P, et al. WHO global antimicrobial resistance surveillance for *Neisseria gonorrhoeae* 2017-18: a retrospective observational study. Lancet Microbe. 2021;2:e627–36. 10.1016/S2666-5247(21)00171-3 35544082

[14] National Academies of Sciences, Engineering, and Medicine; Vermund SH, Geller AB, and Crowley JS, editors. Sexually transmitted infections: adopting a sexual health paradigm. Washington: The National Academies Press; 2021. 10.17226/25955 34432397

[15] Boyer CB, Gaydos CA, Geller AB, Garges EC, Vermund SH. Sexually transmitted infections in the U.S. military: a sexual health paradigm to address risk behaviors, unintended pregnancy, alcohol use, and sexual trauma. Mil Med. 2022;187:140–3. 10.1093/milmed/usab407 34626194 PMC10558038

[16] Sadler AG, Mengeling MA, Syrop CH, Torner JC, Booth BM. Lifetime sexual assault and cervical cytologic abnormalities among military women. J Womens Health (Larchmt). 2011;20:1693–701. 10.1089/jwh.2010.2399 21834691

[17] Garges E, Early J, Waggoner S, Rahman N, Golden D, Agan B, et al. Biomedical response to *Neisseria gonorrhoeae* and other sexually transmitted infections in the US military. Mil Med. 2019;184(Suppl 2):51–8. 10.1093/milmed/usy431 31778198

[18] Tsai AY, Dueger E, Macalino GE, Montano SM, Tilley DH, Mbuchi M, et al. The U.S. military’s *Neisseria gonorrhoeae* resistance surveillance initiatives in selected populations of five countries. MSMR. 2013;20:25–7. 23461308

[19] Sánchez JL, Agan BK, Tsai AY, Macalino GE, Wurapa E, Mbuchi M, et al. Expanded sexually transmitted infection surveillance efforts in the United States military: a time for action. Mil Med. 2013;178:1271–80. 10.7205/MILMED-D-13-00137 24306007

[20] Maiden MC, Bygraves JA, Feil E, Morelli G, Russell JE, Urwin R, et al. Multilocus sequence typing: a portable approach to the identification of clones within populations of pathogenic microorganisms. Proc Natl Acad Sci U S A. 1998;95:3140–5. 10.1073/pnas.95.6.3140 9501229 PMC19708

[21] Kwong JC, Gonçalves da Silva A, Dyet K, Williamson DA, Stinear TP, Howden BP, et al. *NGMASTER:in silico* multi-antigen sequence typing for *Neisseria gonorrhoeae.* Microb Genom. 2016;2:e000076. 10.1099/mgen.0.000076 28348871 PMC5320595

[22] The Clinical and Laboratory Standards Institute. Performance standards for antimicrobial susceptibility testing, 33rd edition. Supplement M100. Wayne (PA): The Institute; 2023.

[23] Centers for Disease Control and Prevention. Gonococcal Isolate Surveillance Project (GISP) and Enhanced GISP (eGISP) protocol [cited 2022 Aug 15]. https://stacks.cdc.gov/view/cdc/125949

[24] Centers for Disease Control and Prevention. Gonococcal isolate surveillance project (GISP) profiles [cited 2024 Jul 22]. https://www.cdc.gov/sti-statistics/gisp-profiles/index.html

[25] Mann LM, Kirkcaldy RD, Papp JR, Torrone EA. Susceptibility of *Neisseria gonorrhoeae* to gentamicin-gonococcal isolate surveillance project, 2015-2016. Sex Transm Dis. 2018;45:96–8. 10.1097/OLQ.0000000000000693 29324629 PMC5861718

[26] Golparian D, Jacobsson S, Holley CL, Shafer WM, Unemo M. High-level in vitro resistance to gentamicin acquired in a stepwise manner in *Neisseria gonorrhoeae.* J Antimicrob Chemother. 2023;78:1769–78. 10.1093/jac/dkad168 37253051 PMC10517096

[27] Holley CL, Dhulipala V, Balthazar JT, Le Van A, Begum AA, Chen SC, et al. A single amino acid substitution in elongation factor G can confer low-level gentamicin resistance in *Neisseria gonorrhoeae.* Antimicrob Agents Chemother. 2022;66:e0025122. 10.1128/aac.00251-22 35465683 PMC9112995

[28] Lyu M, Moseng MA, Reimche JL, Holley CL, Dhulipala V, Su CC, et al. Cryo-EM structures of a gonococcal multidrug efflux pump illuminate a mechanism of drug recognition and resistance. MBio. 2020;11:e00996–20. 10.1128/mBio.00996-20 32457251 PMC7251214

[29] Cartee JC, Joseph SJ, Weston E, Pham CD, Thomas JC IV, Schlanger K, et al. Phylogenomic comparison of *Neisseria gonorrhoeae* causing disseminated gonococcal infections and uncomplicated gonorrhea in Georgia, United States. Open Forum Infect Dis. 2022;9:ofac247. 10.1093/ofid/ofac247 35855008 PMC9280329

[30] World Health Organization. Reporting countries (WHO-GASP) [cited 2020 Nov 12]. https://www.who.int/data/gho/data/indicators/indicator-details/GHO/who-gasp-reporting-countries

[31] Jorge-Berrocal A, Vargas-Herrera N, Benites C, Salazar-Quispe F, Mayta-Barrios M, Barrios-Cárdenas YJ, et al.; Neisseria gonorrhoeae Surveillance Working Group. *Neisseria gonorrhoeae* Surveillance Working Group. Antimicrobial susceptibility of *Neisseria gonorrhoeae* isolates from Peru, 2018 and 2019. Sex Transm Dis. 2022;49:682–6. 10.1097/OLQ.0000000000001678 35858477

[32] Qquellon J, Vargas SK, Eguiluz M, Vasquez F, Durand D, Allan-Blitz LT, et al. Extra-genital *Neisseria gonorrhoeae* infections with genetic mutations conferring ciprofloxacin resistance among men who have sex with men and transgender women in Lima, Peru. Int J STD AIDS. 2023;34:245–50. 10.1177/09564624221147326 36637128 PMC9950594

[33] Sandoval MM, Bardach A, Rojas-Roque C, Alconada T, Gomez JA, Pinto T, et al. Antimicrobial resistance of *Neisseria gonorrhoeae* in Latin American countries: a systematic review. J Antimicrob Chemother. 2023;78:1322–36. 10.1093/jac/dkad071 37192385 PMC10232280

[34] Workneh M, Hamill MM, Kakooza F, Mande E, Wagner J, Mbabazi O, et al. Antimicrobial resistance of *Neisseria gonorrhoeae* in a newly implemented surveillance program in Uganda: surveillance report. JMIR Public Health Surveill. 2020;6:e17009. 10.2196/17009 32519969 PMC7315362

[35] Unemo M, Shafer WM. Antimicrobial resistance in *Neisseria gonorrhoeae* in the 21st century: past, evolution, and future. Clin Microbiol Rev. 2014;27:587–613. 10.1128/CMR.00010-14 24982323 PMC4135894

[36] Butler KR, Lee D, Hollberg M, Posey DL. Overseas gonorrhea screening among newly arrived refugees during 2018. J Immigr Minor Health. 2021;23:1354–8. 10.1007/s10903-021-01270-z 34533683 PMC12631578

[37] Desai AN, Mohareb AM, Hauser N, Abbara A. Antimicrobial resistance and human mobility. Infect Drug Resist. 2022;15:127–33. 10.2147/IDR.S305078 35046676 PMC8763254

[38] Sirivongrangson P, Girdthep N, Sukwicha W, Buasakul P, Tongtoyai J, Weston E, et al.; EGASP Thailand Workgroup. The first year of the global Enhanced Gonococcal Antimicrobial Surveillance Programme (EGASP) in Bangkok, Thailand, 2015-2016. PLoS One. 2018;13:e0206419. 10.1371/journal.pone.0206419 30412586 PMC6226150

[39] Bachmann LH, Barbee LA, Chan P, Reno H, Workowski KA, Hoover K, et al. CDC clinical guidelines on the use of doxycycline postexposure prophylaxis for bacterial sexually transmitted infection prevention, United States, 2024. MMWR Recomm Rep. 2024;73:1–8. 10.15585/mmwr.rr7302a1 38833414 PMC11166373

[40] Molina JM, Charreau I, Chidiac C, Pialoux G, Cua E, Delaugerre C, et al.; ANRS IPERGAY Study Group. Post-exposure prophylaxis with doxycycline to prevent sexually transmitted infections in men who have sex with men: an open-label randomised substudy of the ANRS IPERGAY trial. Lancet Infect Dis. 2018;18:308–17. 10.1016/S1473-3099(17)30725-9 29229440

[41] Bolan RK, Beymer MR, Weiss RE, Flynn RP, Leibowitz AA, Klausner JD. Doxycycline prophylaxis to reduce incident syphilis among HIV-infected men who have sex with men who continue to engage in high-risk sex: a randomized, controlled pilot study. Sex Transm Dis. 2015;42:98–103. 10.1097/OLQ.0000000000000216 25585069 PMC4295649

[42] Luetkemeyer AF, Donnell D, Dombrowski JC, Cohen S, Grabow C, Brown CE, et al.; DoxyPEP Study Team. DoxyPEP Study Team. Postexposure doxycycline to prevent bacterial sexually transmitted infections. N Engl J Med. 2023;388:1296–306. 10.1056/NEJMoa2211934 37018493 PMC10140182

[43] Peters RPH, McIntyre JA, Garrett N, Brink AJ, Celum CL, Bekker LG. Doxycycline post-exposure prophylaxis for sexually transmitted infections in South Africa. South Afr J HIV Med. 2023;24:1510. 10.4102/sajhivmed.v24i1.1510 37795430 PMC10546896

[44] Cehovin A, Jolley KA, Maiden MCJ, Harrison OB, Tang CM. Association of *Neisseria gonorrhoeae* plasmids with distinct lineages and the economic status of their country of origin. J Infect Dis. 2020;222:1826–36. 10.1093/infdis/jiaa003 32163577 PMC7653084

[45] Roberts MC, Knapp JS. Transfer of beta-lactamase plasmids from *Neisseria gonorrhoeae* to *Neisseria meningitidis* and commensal *Neisseria* species by the 25.2-megadalton conjugative plasmid. Antimicrob Agents Chemother. 1988;32:1430–2. 10.1128/AAC.32.9.1430 3143304 PMC175882

[46] Perry CR, Scangarella-Oman NE, Millns H, Flight W, Gatsi S, Jakielaszek C, et al. Efficacy and safety of gepotidacin as treatment of uncomplicated urogenital gonorrhea (EAGLE-1): design of a randomized, comparator-controlled, phase 3 study. Infect Dis Ther. 2023;12:2307–20. 10.1007/s40121-023-00862-6 37751016 PMC10581980

[47] National Institute of Allergy and Infectious Diseases. NIH statement on preliminary efficacy results of first-in-class gonorrhea antibiotic developed through public-private partnership [cited 2023 Nov 1]. https://www.niaid.nih.gov/news-events/nih-statement-preliminary-efficacy-results-first-class-gonorrhea-antibiotic-developed

