# Supplementary material for: Common Patterns and Unique Threats in Antimicrobial Resistance as Demonstrated by Global Gonococcal Surveillance
Source: Emerg Infect Dis. 2024 Nov;30(Suppl 2):S62–70. doi: 10.3201/eid3014.240296 (PMC11559582; doi:10.3201/eid3014.240296)
Supplement: Appendix — Additional information about common patterns and unique threats in antimicrobial resistance as demonstrated by global gonococcal surveillance [file 24-0296-Techapp-s1.pdf]

Article DOI: https://doi.org/10.3201/eid3014.240296

EID cannot ensure accessibility for Supplemental Materials supplied by authors. Readers who have difficulty accessing supplementary content should contact the authors for assistance.

Common Patterns and Unique Threats in Antimicrobial Resistance as Demonstrated by Global Gonococcal Surveillance

Appendix

Appendix Methods

Antimicrobial Susceptibility Testing

Frozen suspensions of NG isolates from five geographic regions were received by the GC Repository, and 962 (77.3%) out of 1244 isolates were confirmed as NG. Isolates that were not confirmed as NG either yielded other bacterial species or did not grow. Minimal inhibitory concentrations (MICs) and whole-genome sequencing was performed for all 962 isolates from the following regions: Eastern Europe: Walter Reed Army Institute of Research (WRAIR) Europe and the Middle East – Tbilisi, Georgia (WRAIR-EME); East Africa: WRAIR-Africa (WRAIR-A), Nairobi & Kisumu, Kenya and Kampala, Uganda; West Africa: Naval Medical Research Unit EURAFCENT- Ghana Detachment – Accra, Ghana; South America: Naval Medical Research Unit SOUTH – Lima, Peru; Asia: Armed Forces Research Institute of Medical Sciences (AFRIMS) – Bangkok & Pattaya, Thailand. MICs were determined for benzylpenicillin, tetracycline, ciprofloxacin, azithromycin, ceftriaxone, cefixime, gentamicin and spectinomycin using ETEST (BioMerieux, Marcy-l'Etoile, France) according to manufacturer's instructions. Agar dilution was performed to confirm MICs for isolates with reduced susceptibility to azithromycin, ceftriaxone, cefixime, and gentamicin. MICs were interpreted according to CLSI guidelines (*1*) or GISP (*2*). AMR testing was repeated for isolates that displayed differences between MICs and identified molecular determinants. Quality control strains used during all AST protocols were: ATCC 49226, WHO K, WHO L and an azithromycin resistant strain obtained from Dr. Olsegun Soge, University of Washington.

The QA program consists of a panel of four control strains used routinely in the GC Repository, plus four clinical isolates collected through GEIS-funded surveillance projects, all of which have been tested by different members of the GC Repository to ensure reproducibility of results.

Whole genome sequencing

DNA was extracted using the DNeasy UltraClean Microbial Kit (Qiagen, Germantown, MD, USA) and libraries constructed using the KAPA Hyperplus Library preparation kit (Roche Diagnostics Indianapolis, Indiana, USA). Libraries were quantified using the KAPA Library Quantification Kit – Illumina/Bio-Rad iCycler (Roche Diagnostics) on a CFX96 real-time cycler (Biorad, Hercules, CA, USA). Libraries were normalized to 1nM, pooled, denatured, and diluted to 20pM. The pooled samples were further diluted to a final concentration of 2.1pM. Samples were sequenced using NextSeq Reagent Kit 500/550 v2 (300 cycle; 2 × 150bp) (Illumina, San Diego, CA, USA. *N. gonorrhoeae* taxonomic classification was confirmed in silico with Kraken2 (*3*) v2.1.2. Further de novo draft genome assemblies were constructed with Shovill v1.1.0 (https://github.com/tseemann/shovill) and coverage statistics were assessed with bbmap v38.96 at minimum thresholds for contig size and coverage set at 200bp and 49.5x, respectively.

Genome analysis

Assemblies were uploaded to SeqSphere+ v8.5.1 (Ridom; Münster, Germany) to assesses genomic similarities across the population based on allele-calling in the *N. gonorrhoeae* core genome (cgMLST) (*4*). A minimum spanning tree (MST) was constructed based on cgMLST allelic differences between all isolates. Neighbor joining trees (NJT) were constructed with SeqSphere+ based on a matrix of pairwise allelic distances. The NJTs were visualized, mid-point rooted, and annotated in iTol v6.8 (*5*).

Identification of genetic determinants of AMR.

Genome-wide analysis was performed to ascertain the presence or absence of genes known to mediate antimicrobial resistance in *N. gonorrhoea*e. All genetic determinants of antimicrobial resistance were identified using both ARIBA v2.14.4 and AMRFinderPlus v3.8.4. Synonymous mutations to the *fusA, gyrA, parC, penA, ponA, porB, rspJ,* and *mtrR* genes and intragenic mutations between *mtrR* and the *mtr*CDE operon were identified by mapping draft

genomes and trimmed reads to wild-type sequences using Minimap2 (*6*) v2.24 within the Geneious Prime v2023.0.4 platform (https://www.geneious.com).

Sequences have been uploaded to Pathogenwatch (https://pathogen.watch/collection/x5yjb93w3z3s-total)

Results

An MST was generated based on cgMLST of all isolates, categorized by geographic location, to reveal both the genomic diversity of the isolates and any clonal lineages (Figure 2 in main article). Isolates from Thailand clustered into 4 major groups, and three appear to be clonal isolates. Georgian isolates were mostly clustered into groups, but a few isolates are closely related to isolates from Thailand (~300 core genome allele differences). Isolates from Peru grouped into 5 clusters. One Peruvian isolate and one Georgian isolate are roughly 130 allelic differences from a Thai isolate, but they do not share typing schemes; they both have novel NG-MAST types, but different NG-STAR and MLST types. However, these three isolates were all resistant to tetracycline, penicillin, ciprofloxacin and have reduced susceptibility to gentamicin.

References

1. Clinical and Laboratory Standards Institute. Performance Standards for Antimicrobial Susceptibility Testing. 33rd ed. CLSI supplement M100. Wayne (PA): The Institute; 2023.

2. Centers for Disease Control and Prevention. Gonococcal Isolate Surveillance Project (GISP) and Enhanced GISP (eGISP) protocol 2020 [cited 2022 Aug 15]. https://www.cdc.gov/std/gisp/GISP_eGISP_Protocol_January_2020.pdf

3. Wood DE, Lu J, Langmead B. Improved metagenomic analysis with Kraken 2. Genome Biol. 2019;20:257. https://doi.org/10.1186/s13059-019-1891-0

4. Harrison OB, Cehovin A, Skett J, Jolley KA, Massari P, Genco CA, et al. *Neisseria gonorrhoeae* population genomics: use of the gonococcal core genome to improve surveillance of antimicrobial resistance. J Infect Dis. 2020;222:1816–25. PubMed https://doi.org/10.1093/infdis/jiaa002

5. Letunic I, Bork P. Interactive Tree Of Life (iTOL) v5: an online tool for phylogenetic tree display and annotation. Nucleic Acids Res. 2021;49(W1):W293–6. PubMed https://doi.org/10.1093/nar/gkab301

6. Li H. New strategies to improve minimap2 alignment accuracy. Bioinformatics. 2021;37:4572–4. PubMed https://doi.org/10.1093/bioinformatics/btab705
